# Effect of prebiotic oligosaccharides on bowel habit and the gut microbiota in children with functional constipation (Inside study): study protocol for a randomised, placebo-controlled, multi-centre trial

**DOI:** 10.1186/s13063-024-08050-8

**Published:** 2024-04-05

**Authors:** Carrie A. M. Wegh, Margriet H. C. Schoterman, Elaine E. Vaughan, Sofie C. C. van der Zalm, Hauke Smidt, Clara Belzer, Marc A. Benninga

**Affiliations:** 1 Department of Paediatric Gastroenterology and Nutrition, Emma Children’s Hospital, Amsterdam UMC, University of Amsterdam, Amsterdam, The Netherlands; 2 Laboratory of Microbiology, Wageningen University & Research, Wageningen, The Netherlands; 3 FrieslandCampina, Amersfoort, The Netherlands; 4 Sensus (Royal Cosun), Roosendaal, The Netherlands

**Keywords:** Gastroenterology, Nutrition, Gut microbiota, Paediatrics, Oligosaccharides, Prebiotic, Functional constipation, Stool consistency, Stool frequency, Bowel habit

## Abstract

**Background:**

Functional constipation (FC) in children is a common gastrointestinal disorder with a worldwide-pooled prevalence of 9.5%. Complaints include infrequent bowel movements, painful defecation due to hard and/or large stools, faecal incontinence, and abdominal pain. Prebiotic oligosaccharides have been shown to relieve constipation symptoms in young adults and elderly. However, sufficient evidence is lacking linking additional prebiotic intake to improve symptoms in children with FC. We hypothesise that prebiotic oligosaccharides are able to relieve symptoms of constipation in young children as well.

**Methods:**

In the present randomised, double-blind, placebo-controlled, multi-centre study, we will study the effects of two prebiotic oligosaccharides in comparison to placebo on constipation symptoms in children of 1–5 years (12 to 72 months) of age diagnosed with FC according to the Rome IV criteria for functional gastrointestinal disorders. The primary outcome measure will be change in stool consistency. Secondary outcomes include stool frequency and stool consistency in a number of cases (%). Tertiary outcomes include among others painful defecation, use of rescue medication, and quality of life. In addition, the impact on gut microbiome outcomes such as faecal microbiota composition and metabolites will be investigated. Participants start with a run-in period, after which they will receive supplements delivered in tins with scoops for 8 weeks, containing one of the two prebiotic oligosaccharides or placebo, followed by a 4-week wash-out period.

**Discussion:**

This randomised double-blind, placebo-controlled multi-centre study will investigate the effectiveness of prebiotic oligosaccharides in children aged 1–5 years with FC.

**Trial registration:**

ClinicalTrials.gov NCT04282551. Registered on 24 February 2020.

**Supplementary Information:**

The online version contains supplementary material available at 10.1186/s13063-024-08050-8.

## Introduction

### Background and rationale

Functional constipation (FC) in children is a common gastrointestinal (GI) disorder with a worldwide prevalence ranging from 0.7 to 29.6%, with a pooled prevalence of 9.5% [1, 2]. Only a minority of patients with FC, both children and adults, seek healthcare [3]. However, it is estimated that up to 25% of visits to a paediatric gastroenterologist are due to FC [4]. Complaints include infrequent bowel movement, painful defecation due to hard and/or large stools, faecal incontinence, and abdominal pain [4]. FC is a clinical diagnosis; the evaluation primarily consists of a thorough medical history and is based on the paediatric diagnostic Rome IV criteria for functional GI disorders (Table 1) [5–7]. Although the condition is rarely life-threatening, it strongly impairs quality of life. The impairment in health-related quality of life is comparable with conditions such as diabetes, rheumatoid arthritis, and chronic allergies [8].

**Table 1:** Rome IV criteria for functional constipation

< 4 years of age [5]	Developmental age of > 4 years [6]
Must include 1 month of at least 2 of the following in infants up to 4 years of age: 1. 2 or fewer defecations per week 2. History of excessive stool retention 3. History of painful or hard bowel movements 4. History of large-diameter stools 5. Presence of a large faecal mass in the rectum In toilet-trained children, the following additional criteria may be used: 6. At least 1 episode/week of incontinence after the acquisition of toileting skills 7. History of large-diameter stools that may obstruct the toilet	Must include 2 or more of the following occurring at least once per week for a minimum of 1 month with insufficient criteria for a diagnosis of irritable bowel syndrome 1. 2 or fewer defecations in the toilet per week in a child of a developmental age of at least 4 years 2. At least 1 episode of faecal incontinence per week 3. History of retentive posturing or excessive volitional stool retention 4. History of painful or hard bowel movements 5. Presence of a large faecal mass in the rectum 6. History of large-diameter stools that can obstruct the toilet After appropriate evaluation, the symptoms cannot be fully explained by another medical condition

The aetiology of FC is still incompletely understood but is likely to be multifactorial. Some factors in children which have been described are withholding behaviour, psychosocial factors such as stressful life events or behavioural problems, behavioural disorders, parental child-rearing attitudes, low fibre intake, and gut microbiota composition [4, 7, 9]. Standard treatment of FC in children includes demystification, education, toilet training, and laxative treatment with, among others, polyethylene glycol (PEG) [10, 11]. Laxatives such as PEG are safe, but adherence to laxatives is low, and except for the use of PEG, little is known about the long-term effects of chronic laxative use [4, 12]. This may explain why 36.4% of parents of children with FC seek help in the form of food supplements and complementary or alternative medicine [13]. One such alternative might be prebiotic oligosaccharides. Galacto-oligosaccharides (GOS) and chicory fructo-oligosaccharides (FOS; synonym oligofructose) are oligosaccharides that belong to the category of prebiotics. Prebiotics are defined by the International Scientific Association for Pro- and Prebiotics (ISAPP) as “a substrate that is selectively utilised by the host microorganisms conferring a health benefit” [14]. GOS and FOS have been shown to selectively stimulate certain gut microbial species, mostly bifidobacteria and lactobacilli, and have demonstrated health benefits, and consequently are endorsed as prebiotics by ISAPP [15–17].

Prebiotic oligosaccharides are of interest due to several factors: (1) low fibre intake has been associated with FC, and oligosaccharides are also considered dietary fibres; (2) fermentation of oligosaccharides is known to increase the abundance of gut microbiota thereby increasing faecal bulk; (3) oligosaccharide-derived microbial fermentation products such as short chain fatty acids (SCFAs) have been described to give energy to colonic epithelial cells and may generate an osmotic effect in the gut, which can increase the water content of faeces, leading to softening of stools; and finally (4), these prebiotics are known to modify the composition of the gut microbiota which may indirectly affect bowel habit via gut-brain signalling. These hypotheses are supported by the fact that prebiotic oligosaccharides have shown stool-softening effects in trials in healthy infants and children with infant, follow-on and young child formulas supplemented with prebiotics [16–25].

In addition, some trials showed improvement in stool consistency in children with FC after the consumption of prebiotic oligosaccharides. However, evidence linking oligosaccharide and/or fibre intake to improved symptoms in children with FC is rather weak [11, 26–30]. This is not only due to the low number of studies, but also the small sample size of studies, overall poor quality of methods used, and incomplete reporting of results. Therefore a large-scale, well-executed study is needed to investigate if the consumption of GOS or FOS can result in improved bowel habits and modify the gut microbiota in young children with FC.

### Objectives

We hypothesise that consumption of GOS or chicory FOS will result in softer stools, improvement of some other constipation-related symptoms and modifications of the gut microbiota in comparison to a placebo. Therefore the aim of the study is to investigate the effect of GOS or FOS versus a placebo on bowel habits and microbiota in children with FC aged 1–5 years.

### Trial design

This is a randomised, double-blind, placebo-controlled, multi-centre trial with three arms: GOS, chicory FOS and placebo, in which GOS will be compared to placebo and FOS will be compared to placebo in a superiority framework.

## Methods

This study (named ‘Inside study’) is a double-blind, randomised, placebo-controlled, multi-centre trial. We aim to enrol 198 children, aged between 1 and 5 years, with FC according to the Rome IV criteria (Table 1).

### Study setting

This study is coordinated by Wageningen University & Research, Laboratory of Microbiology. The study is conducted in The Netherlands. Patients from the outpatient clinics in the Emma Children’s Hospital, Amsterdam, Amsterdam University Medical Centres Amsterdam (AUMC), DeKinderKliniek Almere, Spaarne Gasthuis Haarlem, Haaglanden MC Den Haag, Rijnstate ziekenhuis Arnhem and Maasstad ziekenhuis Rotterdam, will be recruited by their treating paediatric gastroenterologist. More participating centres may follow.

### Eligibility criteria

#### Participant screening

Eligible patients will be contacted by researchers of Wageningen University & Research to answer questions the patients might have and to verify whether or not they are willing to participate, to avoid an undesirable dependency situation with the treating paediatric gastroenterologist.

#### Inclusion criteria

In order to be eligible to participate in this study, a subject must meet all of the following criteria, as considered by a medical doctor:

- Written informed consent obtained from parents or guardians of children meeting the eligibility criteria and those willing to comply with the requirements of the study.
- Aged 1–5 years (12 to 72 months at the day of inclusion).
- Children that meet/fulfil the Rome IV criteria for FC.

#### Exclusion criteria

Any of the following criteria will result in exclusion of a potential subject from this study:

- Children who suffer from any GI complaints other than FC, known structural GI abnormalities, or previous GI surgery.
- Any condition that would make it unsafe for the child to participate. This can include developmental delays associated with musculoskeletal or neurologic conditions affecting the GI tract. Children with an underlying cause of defecation disorder (for example, Hirschsprung’s disease, spina bifida occulta, cystic fibrosis, or GI malformations).
- Children with clinically significant cardiac, vascular, liver, pulmonary, psychiatric disorders, severe renal insufficiency, human immunodeficiency virus, acquired immunodeficiency syndrome, hepatitis B or C or known abnormalities of haematology, urinalysis, or blood biochemistry, as checked by the inclusion questionnaire.
- Children who are lactose intolerant, or who are self-perceived lactose intolerant or for whom it is expected that low doses of lactose could lead to GI symptoms.
- Children who are allergic to cow’s milk or fish.
- Use of antibiotics or other medicines or food supplements, and breast milk-feeding, which can influence defecation and gut microbiota 4 weeks prior to the study run-in period and during the study itself.
- The use of infant formula, follow-on formula, young child formula in the previous week prior to the study run-in period and during the study itself.
- Children on other supplements/medication that could affect bowel function, including e.g. fibre supplements, and pre-, pro- and synbiotics (excluding rescue medication) for the past 4 weeks and during the study itself.
- Children that participate in another clinical trial.

#### Informed consent procedure

Informed consent will be obtained by the researchers or treating paediatric gastroenterologist either at one of the outpatient clinics or at a home visit before the start of the study, see Additional file 1.

### Interventions

**Fig. 1:**
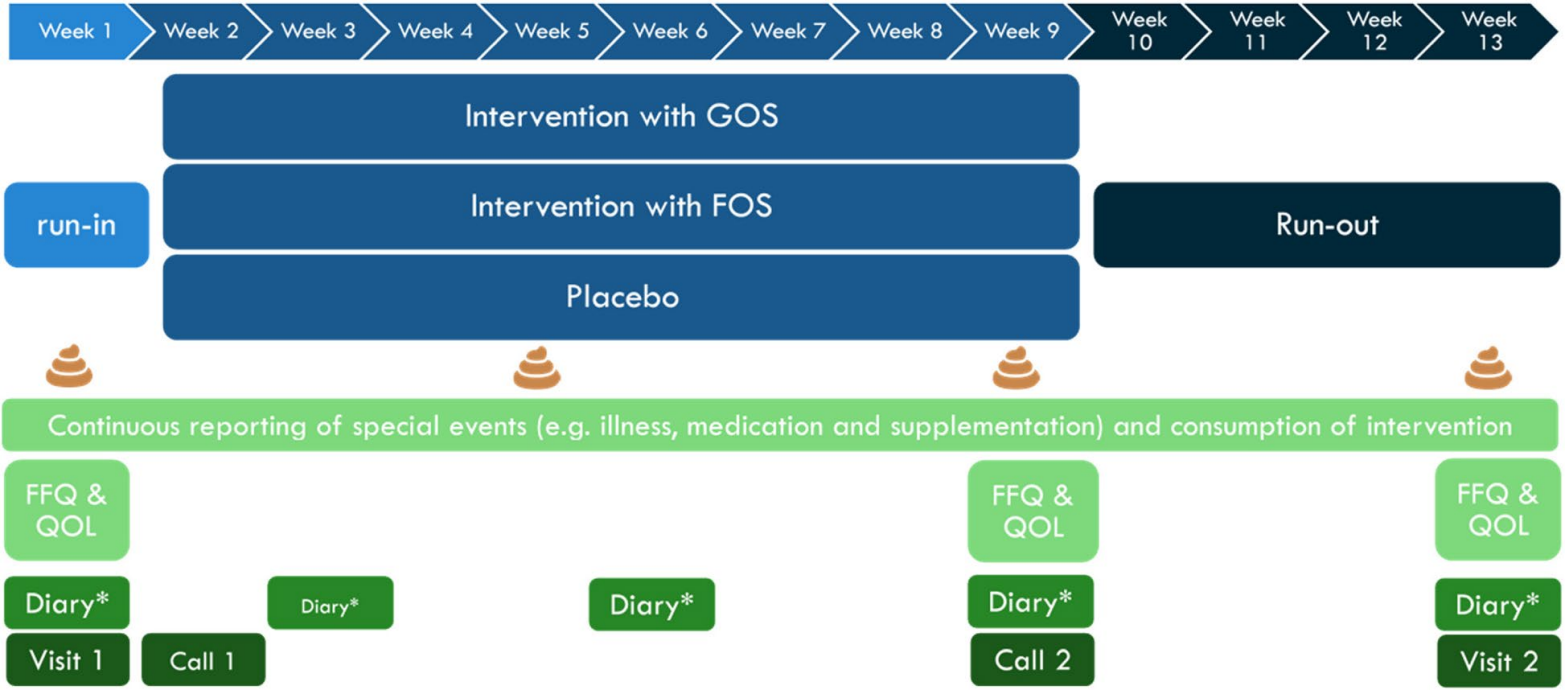
Study protocol flow. Visit 1 is at day = 1, call 1 at day = 7, call 2 at day = 63 (end of 9 weeks), visit 2 at day = 91 (end of 14 weeks), which will be a home visit to pick up samples and leftover product. During visits 1 and 2, anthropometrics (weight, height and head circumference) will be measured. *Diary includes filling out stool consistency and frequency and compliance of taking the study product. FFQ, food frequency questionnaire; FOS, chicory fructo-oligosaccharides; GOS, galacto-oligosaccharides; QOL, quality of life questionnaire

After the run-in period, participants will receive either Vivinal^®^ GOS powder (FrieslandCampina, Amersfoort, The Netherlands), Frutalose^®^ OFP chicory oligofructose (Sensus, Roosendaal, The Netherlands) or placebo (maltodextrin) supplements in tins with scoops. The substances are approved food grade ingredients, they have been previously used in other clinical trials and are used in several food products. All supplements were similarly powders light in colour with a pleasant taste. All supplements are in identical tins with scoops and were produced according to good manufacturing practice standards. One scoop (8.5 mL size) should be consumed per day, with half a dose in the first 3 days to avoid any potential side effects such as flatulence caused by intestinal fermentation of FOS or GOS. The product should preferably be dissolved in warm or cold drinks such as milk or semi-solid products such as yoghurt. The intervention product will be consumed for 8 weeks (Fig. 1).

Rescue medication should be used if the participant does not have a bowel movement for 3 consecutive days, being either microlax 5 mL, sodium picosulphate pearls (1 droplet per 5 kg body mass) or glycerine (glycerol) suppositories (1 g, 2 g or 4 g). These types of laxatives were chosen as they have a mode of action based on provoking peristalsis, and thereby are expected to have minimal effect on gut microbiota composition [31]. This is in contrast to (fermentable) osmotic laxatives such as lactulose or PEG, which were found to influence gut microbiota composition [32].

Despite that rescue medication may be required, the child will still remain in the study. Each use of rescue medication needs to be reported in the diary to differentiate between spontaneous bowel movements and those related to rescue medication use. To further exclude an influence of escape medication on gut microbiota outcomes, a stool sample should only be collected after a spontaneous defecation and at least 3 days after the last use of escape medication.

### Outcomes

#### Primary objective

The main study parameter is the change in stool consistency, measured by the validated Dutch-modified Bristol Stool Form Scale (mBSFS) [33]. This will be the mean difference in stool consistency of GOS versus placebo and FOS versus placebo at all time points (week 1, 3, 6, 9 and 13) and from baseline to week 9.

#### Secondary objectives

The secondary study parameters will be:

- Changes in stool frequency between groups and over time.
- Changes in stool consistency in the number of cases in a certain score of the mBSFS, as percentages.

#### Tertiary objectives

- Painful defecation.
- Meeting the Rome IV criteria at baseline, week 9 and week 13.
- Quality of life of the child, measured by the TAPQOL [34].
- GI symptoms, such as flatulence and bloating.
- Gut microbiome:
  - ◦ Total faecal microbiota composition, as measured by 16S ribosomal RNA (rRNA) gene sequencing
  - ◦ Faecal abundance of specific genera/species as measured by quantitative PCR analysis.
  - ◦ Faecal pH and faecal concentration of fermentation products such as short-chain and branched-chain fatty acids.
  - ◦ Correlations between stool characteristics and gut microbiota composition, faecal pH or fermentation products.
- Use of rescue medication.
- Faecal incontinence (only for completely potty-trained children).
- The amount of GOS, FOS or placebo supplement consumed, as an indication of compliance, measured in both diaries as well as weighing the tins after the trial.
- Anthropometrics: weight, height and head circumference measured at baseline and the close-out visit after week 13.
- Anthropometrics: weight, height and head circumference measured at baseline and the close-out visit after week 13.
- Dietary intake, as measured by a food frequency questionnaire.

### Participant timeline

After randomisation, patients will enter a 1-week run-in period, after which they will either receive GOS, FOS or a placebo for 8 weeks. Lastly, a 4-week run-out period is in place to investigate whether a possible effect lasts or not. The flow of the study protocol is presented in Fig. 1.

### Sample size

A sample size calculation was performed for stool consistency on a scale from 1 to 5. We used the sample size formula *n* = 2 × (Zα + Zβ)2 × (SD/D)2 per group. Using a probability *α* = 0.05 and a power (1-*ß*) of 80%, the formula simplifies to *n* = 2 × 7.9 × (SD/D)2 per group.

The effect sizes of GOS and FOS versus placebo were estimated based on a study by Closa-Monasterolo et al. who investigated the effect of a mix of chicory inulin with FOS on stool consistency in functionally constipated children aged 2–5 years [26]. Based on these data, an effect size of 0.35 was chosen, with an SD of 0.65. This results in a group size of 54.5. The total number of children to be recruited is 198, that is, 66 per arm assuming a drop-out rate of 20%.

### Assignment of interventions: allocation and blinding

Randomisation is done by a computerised random-number generator in the Electronic Data Capture system Castor EDC via a variable block randomisation of block sizes of 6 and 12, not stratified per centre, to one of the three intervention arms [35]. For the study product, two codes per treatment arm, each consisting of two letters and one number were made and printed on the bottom of the cans. The list linking these codes to GOS, FOS or the placebo is only known by two people who are not involved in this study; one at Wageningen University & Research and one at FrieslandCampina. Therefore, the study can be conducted fully blinded for all parties involved. In case of an emergency, the study treatment can be unblinded after consultation with the principal investigator at Wageningen University & Research.

## Data collection and management

### Plans for assessment and collection of outcomes

Data are collected via several means: a diary, weekly report, questionnaires and measurements and clinical symptom reporting during visits. Moreover, parents are asked to collect faecal samples.

#### Diary

The diary is sent daily in the morning via Castor EDC and contains questions on stool frequency and consistency for each defecation, reported for weeks 1, 3, 6, 9 and 13. Moreover, it contains a question on the use of escape medication and on the amount of study product that was consumed for that day. Lastly, in the diary, there is also room for reporting other, not urgent, problems such as mild GI symptoms.

#### Weekly report

The weekly report contains questions on the consumption of the study product (recall) and has room for other, non-urgent, issues such as mild GI symptoms.

#### Questionnaires

Three questionnaires are used in this study. The first one is a general questionnaire, which is only filled out once at the start of the study. This questionnaire includes questions on e.g. duration of breastfeeding and previous antibiotic treatment. Two other questionnaires are a quality of life questionnaire, filled out in weeks 1, 9 and 13. Lastly, to correct for changes in dietary habits, a food frequency questionnaire is filled out in weeks 1, 9 and 13.

#### Clinical symptoms

The Rome IV criteria are confirmed at the inclusion visit and are re-assessed at the end of the intervention period and during the close-out visit.

#### Faecal samples

Parents are asked to collect one faecal sample from weeks 1, 5, 9 and 13 from their child, and store it in a freezer until the close-out visit. Parents are instructed how to collect the sample and are provided with faecal sample tubes with an attached scoop and bags to safely store the sample in their freezer. These tubes are labelled with participant number, duration that the sample was outside of the freezer, date and stool consistency according to the mBSFS. During the close-out visit, faecal samples will be collected and transported on dry ice until they are stored in a -80°C freezer at Wageningen University & Research.

#### Other measurements

To ensure normal growth, anthropometrics (weight, height and head circumference) are measured during the inclusion and close-out visits by the researchers. Besides the reported consumption of the study product in the diaries, tins are weighed before and after the trial for each participant as an additional measure of compliance.

#### Data management and confidentiality

Collected data will be treated confidentially by the study staff associated with the project and according to Regulation (EU) 2016/679 of the European Parliament and of the Council of 27 April 2016 on the protection of natural persons with regard to the processing of personal data and on the free movement of such data, and repealing Directive 95/46/EC (General Data Protection Regulation, in Dutch the Netherlands ‘algemene veroderning gevevensbescherming’ (AVG)) guidelines. Consequently, codes are not based on personal data and are automatically provided by the online system used, i.e. Castor EDC. Data will be reported in electronic case reports (eCRF) and names of the research subjects will be coded, and this code will be used for study products, diaries and questionnaires. The codes list with both the codes and the names of the study participants and other source data will only be accessible to the coordinating investigator and principal investigators, Medical Ethical Committee (METC) and Health Care Inspectorate by a password-protected file in a secured online drive. Interim analyses are not planned.

### Statistical methods

#### Statistical analysis for primary, secondary and tertiary outcomes

Data will be presented as mean ± standard deviation (SD) if normally distributed, or median (interquartile range) when skewed. To test associations between continuous parameters, multiple linear regression will be used. For categorical or dichotomous outcomes, generalised estimation equations or mixed models for repeated measures will be used. Data will be tested for confounding or effect mediators, and confounding factors will be added to the regression. In case of missing data, they will be imputed by multiple imputation or other appropriate methods. All data will be assessed using the statistical program R (The R Foundation for Statistical Computing, Vienna, Austria). A *p*-value of < 0.05 will be considered statistically significant. In order to prevent p-hacking, a false discovery rate correction will be applied for microbiota analyses, of which a *q*-value of < 0.1 is regarded statistically significant. Either GOS or chicory FOS versus the placebo will be analysed for all parameters.

A variety of in-house R-scripts and the Phyloseq package will be used for microbiota composition analyses. To assess variation in microbiota composition, 16S rRNA gene sequence data will be tested for differences between groups in α-diversity (phylogenetic diversity; the number of observed species and inverted Simpson’s for evenness and richness) and β-diversity (Bray–Curtis dissimilarity distances, weighted and unweighted unifrac distances; methods for constrained and unconstrained ordinations; significance of variations by e.g. adonis test). Principal response curves will be used to check the development of the gut microbiota over time. Moreover, an area under the curve assessment for microbiota will be done. For 16S rRNA gene sequence data, a qPCR-based abundance of specific taxa selected based on sequence data and SCFA’s (multiple) linear regression will be used to test the predictive power of the model.

Changes in stool pH, results of the TAPQOL and changes in stool characteristics will be tested by a repeated measure analysis. Differences between timepoints will be assessed using mixed models.

### Monitoring

Data collection, storage and analysis will be the responsibility of the coordinating investigator and principal investigator. The principal investigator will monitor collection, storage and analysis. Moreover, monitoring is planned before enrolment of the first subject, after three subject inclusions, after 60% of intended subjects per site and after the last subject’s last visit. (Serious) Adverse events (SAE/AE) will be monitored throughout the study. In accordance with the legal requirements in the Netherlands (article 10, subsection 1, Medical Research Involving Human Subjects Act (WMO)), the coordinating investigator will inform the subjects and the reviewing accredited METC if harmful events occur. When there are indications that the disadvantage of participation may be significantly greater than was described in the research proposal, the study will be suspended pending a further positive decision by the accredited METC. Moreover, modifications to the study protocol will be reviewed by the METC. Important protocol modifications will be communicated to all relevant parties by the principal investigator.

## Discussion and conclusion

FC is a prevalent problem, especially in young children. Moreover, it ranges from bothersome to have a severe impact on the quality of life of both the child and the family as a whole [36]. At this young age, the children’s diet may be changing as an increasing range of solid foods is introduced to the diet, while simultaneously, mothers’ milk, rich in human milk oligosaccharides (HMOs), or formula milk, usually supplemented with prebiotics, is reduced. Such changes are known to impact bowel habits and the gut microbiota; the young child’s gut microbiota is known to be still rather unstable and less diverse [37]. In addition, the young child will be acquiring the skill of using a potty. Prebiotic oligosaccharides might be a more natural approach to the treatment of FC in children or an additional approach to the conventional treatment of FC in children. Moreover, prebiotic oligosaccharides may play an ameliorating role over the long term via the gut microbiota. However, large-scale, well-executed studies are required to investigate this in children [11, 26–28].

The present study assesses if there is value for prebiotic oligosaccharide consumption in young children with FC. Data from this study will help determine whether children with FC may benefit from consuming prebiotic oligosaccharides such as chicory FOS or GOS. Moreover, both prebiotics are already safely applied in foods as well as in infant, follow-on and young child formulas, hence application should be feasible. GOS, FOS and mixtures thereof have been shown to have stool-softening effects when used in infant, follow-on and young child formulas in healthy infants and in small studies in children with FC [19, 25–27, 29].

Importantly, this is one of the few studies that also gives insight into the gut microbiota of functionally constipated children, besides the impact of prebiotic oligosaccharides on the microbiota. Recently the gut microbiota has been implicated increasingly in not only bowel habit effects but also systemically on metabolism, immunity and the gut-brain axis [38]. Depending on bowel habit outcome effects, it may give some unique insights into the role of the gut microbiota. Moreover, this study will help to further characterise microbial signatures that may be linked to clinical subgroups of children with FC [39]. This might enable us to analyse in detail, which gut microbiota profiles and/or specific microbial populations may be predictive of a positive response, or lack thereof, to treatment with FOS or GOS. Besides the direct symptomatic and microbiological effects, the tertiary outcomes in this study of health-related quality of life and the need for escape medication will provide valuable insight into the perceived wellbeing of children with FC.

Our study has several strengths. First, this study covers many of the clinical outcomes, which are of major interest to clinicians and parents and takes into account most outcomes as suggested by the core outcome set for clinical trials in children with constipation [40]. These include stool consistency and frequency, painful defecation, quality of life of patients and parents, side effects of treatment, and if age-appropriate faecal incontinence [40]. Another strength is that this study also investigates the composition of the gut microbiota, as well as its activity in terms of pH and SCFAs, and the potential role of the gut microbiota in the treatment of FC in children. Moreover, this study also takes into account dietary intake, to also be able to correct for general fibre and fluid intake.

A potential challenge for this study, arising from the nature of the condition, is that many functionally constipated children exert stool-withholding behaviour around the age of toilet training [41]. This behaviour is difficult to address, since the vicious cycle of withholding behaviour, and consequently, the passage of hard and large stools has to be broken. In clinical practice, this cycle is attempted to be broken by giving a higher dose of laxatives, of which the dose is adjusted to the child’s need, due to which a child cannot exert this withholding behaviour. However, this study uses the same dose for all children to first investigate whether prebiotic oligosaccharides may help in the treatment of FC. Within this study, we can differentiate between the children with and without stool-withholding behaviour. However, In case prebiotic oligosaccharides are found to be effective in functionally constipated children, a dose–response study would be a valuable addition to more effectively target those who exert intense stool-withholding behaviour.

A possible limitation of this study is that during this trial current medication use has to be stopped and this might result in (unsatisfactory) changes in bowel habits. Moreover, stopping current treatment and the fact that there is a chance of being in the placebo group might be a disincentive for parents to include their child in this trial. Secondly, the use of rescue medication, despite being selected to have the least influence on gut microbiota composition, will influence bowel habits. However, from the diaries, we can evaluate whether the bowel movement was spontaneous or after the use of rescue medication, although it will be impossible to rule out any potential longer-term influence of the rescue medication on bowel habits. Thirdly, the group of children with FC is very diverse. Therefore it may be a challenge to correct for potential (confounding) factors such as the differences in stool consistency which is known to influence gut microbiota composition, parental child-rearing attitudes and stress factors [42].

Despite these potential limitations, the results of this study could contribute to the development of novel nutritional strategies to support young children with or at risk of FC. It is one of the first studies to thoroughly check the impact of two established prebiotics on FC in children, covering a broad range of parameters. A novel aspect is the additional microbiota composition and activity analyses performed as part of this study. Findings of this study might have important implications for nutrition and supplementation guidelines for children with FC as well as nutritional management of young children with FC which aims to reduce, prevent or treat the symptoms of this condition.

## Trial status

The protocol was approved by the METC of Wageningen University & Research on 21 October 2019 and was registered at ClinicalTrials.gov on 24 February 2020 (NCT04282551). The study protocol was transferred on the 28th of January 2021 to the METC of the Amsterdam Medical Centre (METC AMC) due to the termination of the METC of Wageningen University & Research. Study recruitment started in March 2020. The COVID-19 pandemic resulted in a delay in recruitment and makes it difficult to predict when recruitment will be completed. This current published protocol which was improved with minor amendments is protocol version 7.2 (06–01-2023).

### Supplementary Information

**Additional file 1.** informed consent.

## Data Availability

Data will be stored in a data repository and human material will be stored for 5 years after completion of the study at the sponsor’s site. Participant data will be stored digitally and kept for 15 years at the sponsor’s site and 2 years at the participating hospital. Faecal samples will be stored for 5 years after completion of the study at the sponsor’s site. Participants are asked in the informed consent form whether they provide consent to also use these samples for potential future studies.

## Abbreviations

AE: Adverse event
FC: Functional constipation
FOS: Fructo-oligosaccharides
GI: Gastrointestinal
GOS: Galacto-oligosaccharides
HMO: Human milk oligosaccharides
ISAPP: International Scientific Association for Pro- and Prebiotics
mBSFS: Modified Bristol Stool Form Scale
METC: Medical ethical reviewing committee
PEG: Polyethylene glycol
qPCR: Quantitative polymerase chain reaction
rRNA: Ribosomal ribonucleic acid
SAE: Serious adverse event
SD: Standard deviation
V: Visit
WMO: Medical Research Involving Human Subjects Act
