# Supplementary material for: Effect of prebiotic oligosaccharides on bowel habit and the gut microbiota in children with functional constipation (Inside study): study protocol for a randomised, placebo-controlled, multi-centre trial
Source: Trials. 2024 Apr 5;25:238. doi: 10.1186/s13063-024-08050-8 (PMC10996211; doi:10.1186/s13063-024-08050-8)
Supplement: Supplementary file 1 — Additional file 1. informed consent. [file 13063_2024_8050_MOESM1_ESM.pdf]

Bijlage C: toestemmingsformulier proefpersoon

Prebiotische vezels of een placebo bij kinderen met functionele obstipatie

Ik ben gevraagd om toestemming te geven voor deelname van de mijn kind aan dit medisch-wetenschappelijke onderzoek:

Naam proefpersoon (kind): Geboortedatum: __ / __ / __

- Ik heb de informatiebrief voor de proefpersoon/ouders verzorgers gelezen. Ook kon ik vragen stellen. Mijn vragen zijn voldoende beantwoord. Ik had genoeg tijd om te beslissen of ik wil dat mijn kind meedoet.
- Ik weet dat meedoen vrijwillig is. Ook weet ik dat ik op ieder moment kan beslissen dat mijn kind toch niet mee doet. Daarvoor hoef ik geen reden te geven.
- Ik geef toestemming voor het informeren van de specialist(en) die mijn kind behandelt dat mijn kind meedoet aan dit onderzoek.
- Ik geef toestemming voor het verzamelen en gebruiken van de gegevens en lichaamsmateriaal van mijn kind voor de beantwoording van de onderzoeksvraag in dit onderzoek. Ik weet dat persoonsgegevens 15 jaar moeten worden bewaard.
- Ik weet dat voor de controle van het onderzoek sommige mensen toegang tot alle gegevens van mijn kind kunnen krijgen. Die mensen staan vermeld in deze informatiebrief. Ik geef toestemming voor die inzage door deze personen.
- Ik geef toestemming voor het informeren van de huisarts en/of behandelend specialist van mijn kind over onverwachte bevindingen die van belang (kunnen) zijn voor de gezondheid van mijn kind.
- Ik geef □ **wel**

  □ **geen**

  toestemming om het lichaamsmateriaal van mijn kind na dit onderzoek 5 jaar te bewaren en om dit later nog voor meer onderzoek te gebruiken, zoals in de informatiebrief staat.
- Ik geef □ **wel**

  □ **geen**

  toestemming om mij opnieuw te benaderen na dit onderzoek voor een vervolgonderzoek bij mijn kind.
- Ik wil □ **wel**

  □ **niet**

  geïnformeerd worden over welke behandeling mijn kind heeft gehad/in welke groep mijn kind zat.
- Ik ga ermee akkoord dat mijn kind meedoet aan dit onderzoek.

Naam ouder/voogd**:
Handtekening: Datum: __ / __ / __
Naam ouder/voogd**:
Handtekening: Datum: __ / __ / __

Ik verklaar hierbij dat ik bovengenoemde persoon/personen volledig heb geïnformeerd over het genoemde onderzoek.

Als er tijdens het onderzoek informatie bekend wordt die de toestemming van de ouder of voogd zou kunnen beïnvloeden, dan breng ik hem/haar daarvan tijdig op de hoogte.

Naam onderzoeker (of diens vertegenwoordiger):
Handtekening: Datum: __ / __ / __

Aanvullende informatie is gegeven door:
Naam:
Functie:
Handtekening Datum: __ / __ / __

** Als het kind jonger dan 16 jaar is, ondertekenen de ouders die het gezag uitoefenen of de voogd dit formulier.
